# Rapid testicular metastasis from bladder transitional urothelial carcinoma after radical cystoprostatectomy: A case report

**DOI:** 10.1097/MD.0000000000018000

**Published:** 2019-11-27

**Authors:** Kaijie Wu, Junjie Fan, Hua Liang, Yu Yao, Dalin He

**Affiliations:** a Department of Urology; b Department of Pathology; c Department of Medical Oncology, First Affiliated Hospital of Xi’an Jiaotong University, Xi’an, P.R. China.

**Keywords:** bladder cancer, metastasis, testicular cancer

## Abstract

**Rationale:**

Bladder cancer (BC) is commonly diagnosed in the urinary system and the most common subtype is transitional urothelial carcinoma (TCC). Even with the best treatment, tumor recurrence and metastases always occur. While clinicians commonly observe the metastases to pelvic lymph nodes, liver, lung, and bone, it may infrequently spread to some uncommon locations.

**Patient concerns:**

The patient was a 67-year-old man with a diagnosis of high-grade TCC with squamous differentiation in the bladder and prostate. Subsequently, radical cystoprostatectomy, adjuvant radiotherapy, and chemotherapy were performed. However, he felt intermittent right scrotal pain about 1 year later.

**Diagnosis:**

Ultrasound strongly suggested a testicular neoplasm of right testis, but the left was normal.

**Interventions:**

The patient underwent a right radical orchiectomy and histopathology confirmed testicular metastatic neoplasm from bladder. Moreover, further examination with positron emission tomography revealed no visible distant spread of the urothelial carcinoma.

**Outcomes:**

No signs of tumor recurrence or distant metastasis were visible under follow-up 1 year after radical orchiectomy.

**Lessons:**

Testicular mass may be metastatic tumor during follow-up for patients who were diagnosed as BC, especially for TCC with variant histology. The reason of this could be explained of residual micrometastases after surgery and need more examination to discover local micrometastases to apply more aggressive treatment.

## 1 Introduction

Transitional urothelial carcinoma (TCC) is the most common subtype of bladder cancer (BC). TCC with variant histology is associated with a higher grade and stage, and it may metastasize more aggressively.^[1]^ Most testicular metastases are commonly incidentally found at autopsy, and the diagnosis mainly depends on histopathology. In addition to prostate, kidney, and other common primary lesions, the bladder should not be overlooked.^[2]^ Metastatic tumors of the testis is rare, especially secondary to BC and even more rare 1 year after surgery. Only 9 patients with BC testicular metastases have been reported from 1994 to date. Moreover, all of them chosen 1 or 2 of surgery, radiotherapy, chemotherapy as initially treatment for BC. Herein, we describe the first case of the patient who underwent radical cystoprostatectomy, adjuvant radiotherapy, and chemotherapy presented with emerging rapid testicular metastases without distant metastases.

## 2 Case presentation

Owing to dysuria, a 67-year-old male patient underwent transurethral resection of prostate in February 2017. During the operation, a mass was found in the bladder neck, and histopathology showed a high-grade TCC with squamous differentiation in the bladder and prostate.

He then underwent a robot-assisted radical cystoprostatectomy plus urethrectomy, pelvic lymphadenectomy, seminal vesicle dissection, urethral cavernous dissection, and ileal conduit in May 2017. The operation was uneventful, and the patient was discharged 8 days later. Subsequently, histopathologic examination of the surgical specimens revealed a high-grade TCC with squamous differentiation of the bladder and prostate, extensively invading the capsula prostatica, bilateral seminal vesicle, and right pelvic lymph node (2/3 positive), and the formation of intravascular thrombosis was noted. The 4 left pelvic lymph nodes, the resection margins and corpus spongiosum were TCC-negative. After the operation, the patient immediately received adjuvant radiotherapy plus 4 cycles of oxaliplatin combined with gemcitabine adjuvant chemotherapy, which were initially considered sufficient.

**Figure 1:**
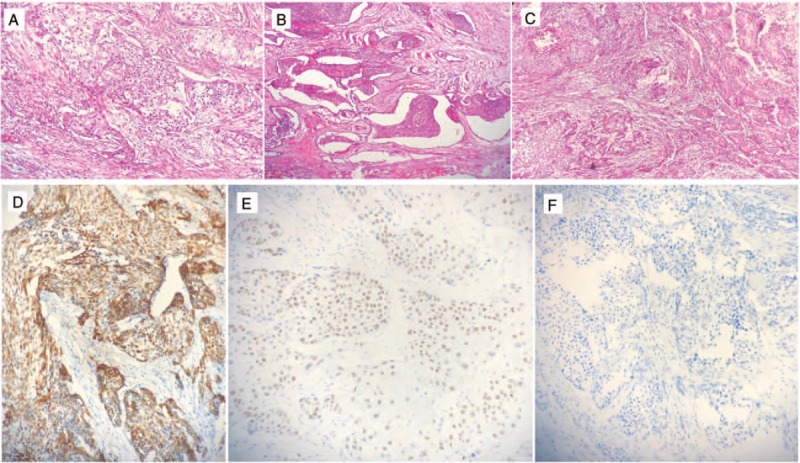
(A) Hematoxylin and eosin staining showed that urothelial carcinoma metastases in the right testis were arranged as a nest and (B) invaded the testicular reticulum and (C) seminiferous tube. Three micrographs illustrate (D) CK5/6^+^, (E) P63^+^, (F) GATA3^−^ tumor cells.

**Figure 2:**
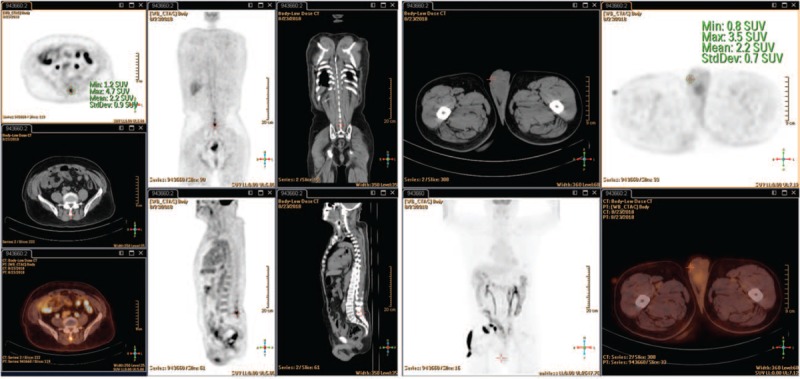
Positron emission tomography with fluorine-18 fluorodeoxyglucose integrated with computed tomography showed no evidence of distant metastases and locoregional failure of the urothelial carcinoma.

A regular follow-up showed no evidence of tumor recurrences until August 2018. He presented with a 2-month history of intermittent right scrotal pain. The ultrasound strongly suggested a testicular neoplasm of right testis, but the left testis was normal. Then, a right radical orchiectomy was performed, and histopathology showed a metastatic poorly differentiated carcinoma with necrosis. Upon microscopic examination, metastatic tumor cells in testis were arranged as a nest and invaded the testicular reticulum and seminiferous tube. An immunostaining panel revealed that the tumor cells were positive for CK5/6, P63 and CD34 but negative for CK20, CK7, GATA3, uroplakin, and D2-40. The histologic characteristics and immunohistochemical profile were consistent with the metastasis of a high-grade TCC from the bladder (Fig. 1). After the orchiectomy, positron emission tomography with fluorine-18 fluorodeoxyglucose integrated with computed tomography was performed and did not reveal any visible spread of the urothelial carcinoma (Fig. 2). Under follow-up for 1 year, he still performed well and had no evidence of recurrence or distant metastasis.

## 3 Discussion

BC is among the most common cancers worldwide, with approximately 81,000 new cases diagnosed in USA in 2018; BC is histologically categorized into TCC, squamous cell carcinoma, adenocarcinoma, and so on.^[3,4]^ TCC with a higher grade and stage may metastasize more aggressively, especially TCC with variant histology.

The common sites of metastasis from TCC of the bladder, in order of decreasing frequency, are the pelvic lymph nodes, liver, lung, and bone.^[5]^ Owing to the blood-testis barrier and lower temperatures in the scrotum, metastasis to the testis is extremely rare, especially for bladder tumors.^[6]^ However, a review of 425 men who underwent cystoprostatectomy showed that an indirect spread to the prostate and surrounding tissues was more common than ever thought.^[7]^ Therefore, urologists should pay attention to a possibility of metastatic carcinoma when a new testicular mass was found in patients with high-grade TCC.

Radical cystoprostatectomy (RC) is a standard, potentially curative, surgical treatment for patients with muscle-invasive BC (MIBC) or high-risk non-muscle-invasive BC. However, patients with the local advanced BC (pT3-pT4, N- or N+, M0) have a high mortality rate after RC, especially for N+ BC.^[8]^ Therefore, the European Association of Urology guidelines recommend adjuvant radiotherapy as an option for local advanced BC to decrease local recurrence.^[9]^ Mohamed et al analyzed the characteristics of 120 patients who received adjuvant radiotherapy plus chemotherapy or chemotherapy alone; this study revealed that the combination of adjuvant radiotherapy and chemotherapy was associated with a significant improvement in the local recurrence-free survival and marginal improvements in the disease-free survival.^[10]^ Moreover, Ymir et al reported that the adjuvant chemotherapy after radical cystoprostatectomy could lead to a 10-year recurrence-free follow-up period despite the presence of testicular micrometastases and involvement of the blood-testis barrier.^[11]^ Therefore, based on the existing literature, we expected that the adjuvant radiotherapy plus chemotherapy combination after operation would improve the survival rate of patients. However, in our case, despite adjuvant radiotherapy and 4 cycles chemotherapy given after RC, this patient, unfortunately, had testicular metastases in less than a year. In addition to considering poor differentiation of the tumor and the wide range of invasion, other factors that promote metastatic tumor growth need to be discussed.

The diffusion of BC cells can be divided into the following routes:

(1) direct invasion;
(2) hematogenous pathway;
(3) lymphatic pathway;
(4) implantation metastasis.

Thomas et al analyzed 26 cases of nonincidental carcinoma metastasis to the testis and found that prostate is the most common primary site. They also noted that tumor cells may spread through the prostatic ducts or lymphatic pathway to the testis.^[2]^ Recently, in a case report by Ymir et al, TCC of the bladder spread to the testis not only by prostatic ducts but also through the vas deferens.^[11]^ Therefore, we assume that the main metastatic pathway from TCC of the bladder to testes is by endovascular growth or the lymphatic pathway. Because our histopathology revealed that the tumor cells invaded the capsula prostatica, bilateral seminal vesicle, and right pelvic lymph node, and tumor cells also formed the intravascular thrombosis, it was possible that there were testicular micrometastases at that time. Moreover, a review by Marlon et al highlighted that the extended pelvic lymph node dissection templates seemed to provide an optimal recurrence-free and cancer-specific survival, and the increased nodal yield improved the oncological outcomes in patients with node-negative or node-positive disease.^[12]^ In addition, Morgan et al found a compromised overall mortality when 1–5 nodes (hazard ratio [HR] 1.33, 95% confidence interval [CI]: 1.12–1.58) or 6–13 nodes (HR 1.18, 95% CI: 1.00–1.40) were harvested compared with a yield of >14 nodes.^[13]^ In our case, only 7 pelvic lymph nodes were dissected during operation, and it was also possible to have residual lymph nodes metastases at that time.

In this case, identification of urothelial carcinoma metastases in the testicle was aided by immunohistochemical staining for the CK5/6 protein. CK5/6 is a basal cytokeratin, which is expressed in urothelial carcinoma and positivity signifies adverse prognostic features such as high-grade and muscularis propria invasion.^[14]^ Moreover, Woo et al utilized whole-genome mRNA expression profiling and hierarchical cluster analysis to show that the basal MIBC were significantly enriched with squamous features and expressed basal biomarker mRNA, such as CK5/6, at higher levels. Additionally, MIBC enriched with the basal-type proteins CK5/6 showed a significantly worse prognosis.^[15]^ This was consistent with the characteristics of our case. Taken together, all of these might explain why this patient had a rapid testicular metastasis after surgery, adjuvant radiotherapy and chemotherapy. Therefore, for patients with aggressive malignancy and a wide range of invasion, the need for further examination after radical surgery remains to be explored.

Attention should be paid to the possibility of metastatic carcinoma when a new testicular mass is found, especially in patients with the basal subtype of BC, and further examination after radical surgery should be performed to discover local residual micrometastases.

## Acknowledgments

The authors thank Rong Wang, Department of Radiology, for her help with the preparation of the radiological materials. We also thank Guanjun Zhang, Department of Pathology, for his help in this paper.

## Author contributions

**Conceptualization:** Dalin He.

**Data curation:** Junjie Fan.

**Formal analysis:** Kaijie Wu, Junjie Fan.

**Methodology:** Hua Liang.

**Project administration:** Yu Yao.

**Writing – original draft:** Kaijie Wu, Junjie Fan.

**Writing – review and editing:** Yu Yao, Dalin He.
